# Association of Peripheral Inflammatory Indices With Symptom Severity, Functioning, and Metabolic Parameters in the Mexican Population Diagnosed With Schizophrenia

**DOI:** 10.7759/cureus.106527

**Published:** 2026-04-06

**Authors:** Héctor Guzmán-González, Ricardo Bernal-Santos, Claudia Morales-Martínez, Mauricio Rosel-Vales

**Affiliations:** 1 Schizophrenia Clinic, Instituto Nacional de Psiquiatría Ramón de la Fuente Muñiz, Mexico City, MEX; 2 Department of Education, Instituto Nacional de Psiquiatría Ramón de la Fuente Muñiz, Mexico City, MEX

**Keywords:** inflammation, metabolism, peripheral inflammatory indices, psychosis, schizophrenia

## Abstract

Introduction: Schizophrenia is a chronic psychotic disorder characterized by systemic immune activation and significant psychosocial impairment. While peripheral inflammatory indices (PIIs) are cost-effective and accessible markers of systemic inflammation, their relationship with clinical and metabolic parameters remains scarce in the Mexican population. This study aims to evaluate the association between PIIs and symptom severity, functioning, and metabolic parameters in patients with schizophrenia under routine clinical care.

Methods: The sample comprised 100 clinically stable patients diagnosed with schizophrenia. Participants underwent a clinical interview and were assessed using the Positive and Negative Syndrome Scale (PANSS), the Functioning Assessment Short Test (FAST), and the Calgary Depression Scale for Schizophrenia (CDSS). Laboratory data from the preceding 12 months were used to calculate the following PIIs: neutrophil-to-lymphocyte ratio (NLR), monocyte-to-lymphocyte ratio (MLR), platelet-to-lymphocyte ratio (PLR), systemic immune-inflammation index (SII), systemic inflammation response index (SIRI), and aggregate index of systemic inflammation (AISI). Associations were analyzed using Spearman's partial rank correlations, adjusting for body mass index, age, olanzapine equivalents, and smoking status.

Results: No significant correlations were found between PIIs and PANSS, FAST, or CDSS scores. Weak positive correlations were observed between glycated hemoglobin (HbA1c) and SII (*r_s_* (98) = 0.242, *p* = 0.017), AISI (*r_s_* (98) = 0.256, *p* = 0.012), and SIRI (*r_s_* (98) = 0.229, *p* = 0.025). In addition, triglycerides showed weak negative correlations with NLR(*r_s_* (98) = -0.246, *p* = 0.016), MLR (*r_s_* (98) = -0.260, *p* = 0.011), PLR (*r_s_* (98) = -0.256, *p* = 0.012), and SII (*r_s_* (98) = -0.219, *p* = 0.032).

Conclusion: Our findings suggest that in clinically stable patients, PIIs are associated with metabolic impairment, specifically glycemic and triglyceride alterations, rather than with symptom severity or functioning. These indices may serve as accessible immunometabolic indicators in this population.

## Introduction

Schizophrenia is a chronic psychotic disorder with a lifetime prevalence of 0.4%, typically emerging during early adulthood and involving significant psychosocial impairment [1]. This disorder is characterized by the presence of positive symptoms (such as delusions, hallucinations, and disorganized speech and behavior), negative symptoms (including affective blunting, alogia, anhedonia, avolition, and social withdrawal), and cognitive impairments [2]. Although traditional pharmacological treatments primarily focus on dopaminergic modulation, approximately 30% of patients remain treatment-resistant [3]. This suggests alternative pathophysiological mechanisms, including immunological changes mediated by inflammation.

Elevated concentrations of serum cytokines (including IL-1, IL-6, and TNF-α) and complement proteins (such as C3, C3b, and C4), along with alterations in the distribution of lymphocyte subpopulations in patients diagnosed with schizophrenia, support the perspective of this disorder as a proinflammatory condition [4-6]. Nevertheless, the high costs and technical requirements for quantifying these markers limit their use in routine clinical practice in Mexico. Consequently, peripheral inflammatory indices (PIIs) have emerged as cost-effective and accessible measures of inflammation, as they can be calculated from standard complete blood counts (CBCs) [7]. These indices have demonstrated significant predictive value in various clinical contexts, including sepsis and oncological diseases [8].

Among PIIs, the neutrophil-to-lymphocyte ratio (NLR) has been reported to be elevated in patients during the acute phase of schizophrenia compared to control groups, indicating an inflammatory process characterized by a predominance of neutrophils in peripheral blood [9]. Furthermore, higher NLR values have been linked with the severity of positive and negative symptoms, cognitive impairment, and aggressive behavior [10-13]. Conversely, the reduction of NLR following antipsychotic initiation suggests that clinical stabilization may be partially mediated by the immunomodulatory effects of these medications [14]. Regarding metabolic alterations, one study identified the platelet-to-lymphocyte ratio (PLR) as a potential indicator of metabolic syndrome in schizophrenia, which is important given the high prevalence of cardiometabolic comorbidities in these patients [15].

To date, no published research has investigated the association between PIIs and symptom severity, psychosocial functioning, or metabolic variables within the Mexican population. Utilizing a pragmatic and exploratory study design, we aimed to evaluate the relationship between these parameters in clinically stable patients under routine clinical care. We hypothesize that higher inflammatory indices will be associated with increased symptom severity, metabolic dysregulation, and diminished functioning.

## Materials and methods

Participants

This study was conducted between September 2025 and February 2026 at the Schizophrenia Clinic of the Instituto Nacional de Psiquiatría in Mexico City. Using a convenience sampling, we recruited outpatients aged ≥18 years with a diagnosis of schizophrenia according to the International Classification of Diseases, 11th Revision (ICD-11) [16]. Participants were clinically stable patients receiving antipsychotic treatment and with no psychiatric hospitalization within the previous year. Patients treated with clozapine were excluded to avoid potential treatment-induced hematological alterations. Additional exclusion criteria included a history of autoimmune, hematological, or oncological diseases, known immunodeficiencies, or the use of related therapies.

Study procedures

A trained psychiatrist conducted a clinical interview to collect sociodemographic and clinical data, including blood pressure, weight, and height. The following instruments were administered: the Positive and Negative Syndrome Scale (PANSS), the Functioning Assessment Short Test (FAST), and the Calgary Depression Scale for Schizophrenia (CDSS). Laboratory data from the preceding 12 months were retrieved from electronic medical records, including CBC, lipid profile, fasting glucose, and glycated hemoglobin (HbA1c). These tests are performed for all patients annually as part of the clinic's standard protocol.

Sample collection and analysis

Blood samples were collected under standardized conditions (overnight fast and venipuncture between 7:00 and 9:00 hours). For the CBC and HbA1c analysis, blood was drawn into ethylenediaminetetraacetic acid (EDTA)-containing Vacutainer tubes. While the CBC was processed using a Mindray BC-760 automated hematology analyzer (Shenzhen, China), HbA1c was determined using the Vitros 4600 (Ortho Clinical Diagnostics, Raritan, NJ, USA) chemistry system. Additionally, fasting glucose and lipid profile were analyzed on the same Vitros 4600 system using samples collected in clot activator tubes. Based on these parameters, the following PIIs were calculated: NLR (neutrophils/lymphocytes), monocyte-to-lymphocyte ratio (MLR, monocytes/lymphocytes), PLR (platelets/lymphocytes), systemic immune-inflammation index (SII, (platelets x neutrophils)/lymphocytes), systemic inflammation response index (SIRI, (monocytes x neutrophils)/lymphocytes), and aggregate index of systemic inflammation (AISI, (platelets x monocytes x neutrophils)/lymphocytes).

Symptom severity

Symptom severity was assessed using the PANSS, a 30-item instrument. Each item is rated on a 7-point Likert scale, where 1 indicates the absence of the symptom, and 7 indicates extreme severity. The total score ranges from 30 to 210, with higher scores indicating greater severity [17]. In the Mexican population, the five-factor model (positive, negative, cognitive, excitement, and anxiety/depression factors) demonstrated high internal consistency, with Cronbach’s alpha coefficients ranging from 0.80 to 0.89 across factors [18]. The authors obtained the necessary license for the PANSS through the MAPI Research Trust ePROVIDE platform.

Functional assessment

The FAST was used to evaluate functional impairment. This scale is a 24-item instrument that evaluates six domains of functioning: autonomy, occupational functioning, cognitive functioning, financial issues, interpersonal relationships, and leisure time. Each item is rated on a 4-point Likert scale ranging from 0 (no difficulty) to 3 (severe difficulty). The total score ranges from 0 to 72, with higher scores reflecting greater functional impairment [19]. The Spanish version has shown high internal consistency in patients with schizophrenia (Cronbach’s alpha of 0.87) [20].

Depression screening

Depressive symptomatology was measured using the CDSS, a nine-item instrument specifically designed for schizophrenia. Each item is scored on a 4-point Likert scale from 0 (absent) to 3 (severe). The total score ranges from 0 to 27, with higher scores indicating greater depressive symptomatology [21]. The Spanish version showed good internal consistency (Cronbach’s alpha of 0.83), with a validated cutoff point of 5 for clinical depression [22]. The authors obtained the necessary license for the CDSS through the MAPI Research Trust ePROVIDE platform.

Statistical analysis

Categorical variables were represented by frequencies and percentages. Data normality was assessed using the Kolmogorov-Smirnov test. Since the results indicated a non-normal distribution, continuous variables were reported as medians with interquartile ranges (IQRs). Associations between variables were analyzed using Spearman's partial rank correlations, adjusting for body mass index (BMI), age, olanzapine equivalents, and smoking status. A significance level of *p* < 0.05 was established. All statistical analyses were performed using Jamovi (version 2.7.23.0) for macOS.

Ethical considerations

The study was conducted in accordance with the principles of the Declaration of Helsinki and was approved by the Ethics Committee of the Instituto Nacional de Psiquiatría under approval number CEI/C/036/2025. All participants provided written informed consent prior to their participation in the research. To safeguard confidentiality, personal data was recorded using an alphanumeric code. The authors declare no conflicts of interest.

## Results

Sociodemographic and clinical characteristics

The study included 100 participants, of whom 66 (66.00%) were men. The median age was 44.00 (IQR = 19.75) years. Among them, 17 (17.00%) participants were in a relationship, and 37 (37.00%) were employed. The median education was 11.00 (IQR = 5.00) years.

The median age at disorder onset was 23.50 (IQR = 11.25) years, with a median illness duration of 17.00 (IQR = 18.25) years. Participants maintained a stable antipsychotic dosage for a median of 36.00 (IQR = 63.00) months, with a median olanzapine-equivalent dose of 15.00 (IQR = 10.00) mg/day. Additional characteristics are detailed in Table 1.

**Table 1:** Sociodemographic and clinical characteristics. BMI: body mass index; DBP: diastolic blood pressure; IQR: interquartile range; SBP: systolic blood pressure. *Long-acting injectable antipsychotics.

	Median	IQR
Age (years)	44.00	19.75
Education (years)	11.00	5.00
Age at onset (years)	23.50	11.25
Disorder duration (years)	17.00	18.25
Time of stable antipsychotic dosage (months)	36.00	63.00
Olanzapine equivalents (mg/day)	15.00	10.00
Hospitalizations	1.00	1.00
BMI (kg/m^2^)	29.81	7.20
SBP (mmHg)	114.00	23.00
DBP (mmHg)	72.50	20.00
	n	%
Sex (men)	66	66
Employment	37	37
Relationship	17	17
Tobacco use	35	35
Diabetes mellitus	22	22
Systemic arterial hypertension	14	14
Lipid-lowering agents therapy	12	12
Glucose-lowering medications	21	21
Antihypertensive agents	12	12
Antidepressant therapy	27	27
Aripiprazol	23	23
Olanzapine	39	39
Risperidone	16	16
Paliperidone*	8	8
Quetiapine	2	2
Sulpiride	2	2
Haloperidol	7	7
Flupentixol*	1	1
Zuclopentixol*	1	1
Trifluoperazine	1	1

Clinical assessment

Median scores indicated moderate symptom severity (PANSS: 81.00, IQR = 32.00) and moderate functional impairment (FAST: 40.50, IQR = 21.50). The median CDSS score was 2.00 (IQR = 5.25). Using a cutoff point of ≥5, 30 (30.00%) patients screened positive for a depressive episode. Detailed scores of each scale are presented in Table 2.

**Table 2:** Clinical rating scales and laboratory parameters. AISI: aggregate index of systemic inflammation; CDSS: Calgary Depression Scale for Schizophrenia; FAST: Functioning Assessment Short Test; HbA1c: glycated hemoglobin; HDL-C: high-density lipoprotein cholesterol; IQR: interquartile range; MLR: monocyte-to-lymphocyte ratio; NLR: neutrophil-to-lymphocyte ratio; PANSS: Positive and Negative Syndrome Scale; PLR: platelet-to-lymphocyte ratio; SII: systemic immune-inflammation index; SIRI: systemic inflammation response index.

	Median	IQR
PANSS		
Positive	24.00	9.25
Negative	25.00	10.50
Cognitive	16.00	7.00
Excitement	7.00	6.00
Anxiety/depression	8.00	5.00
Total	81.00	32.00
FAST		
Autonomy	7.00	5.00
Occupational functioning	11.00	10.00
Cognitive functioning	6.00	5.00
Financial	3.00	5.00
Interpersonal relationships	9.00	6.25
Leisure time	4.00	3.00
Total	40.50	21.50
CDSS	2.00	5.25
Neutrophils (10^3^/μL)	3.95	1.81
Lymphocytes (10^3^/μL)	2.08	1.12
Monocytes (10^3^/μL)	0.39	0.19
Platelets (10^3^/μL)	230.00	81.25
Glucose (mg/dL)	93.50	12.00
HbA1c (%)	5.40	0.90
Triglycerides (mg/dL)	162.74	82.00
Cholesterol (mg/dL)	186.50	55.25
HDL-C (mg/dL)	44.50	16.94
NLR	1.80	0.85
PLR	101.49	50.86
MLR	0.18	0.08
SII	413.83	250.85
SIRI	0.69	0.55
AISI	170.28	159.39

Peripheral inflammatory indices

The median values of the PIIs were the following: NLR, 1.80 (IQR = 0.85); PLR, 101.49 (IQR = 50.86); MLR, 0.18 (IQR = 0.08); SII, 413.83 (IQR = 250.85); AISI, 170.28 (IQR = 159.39); and SIRI, 0.69 (IQR = 0.55). 

Metabolic parameters

Regarding metabolic parameters, the median values were the following: fasting glucose 93.50 (IQR = 12.00) mg/dL; HbA1c, 5.40% (IQR = 0.90); triglycerides, 162.74 (IQR = 82.00) mg/dL; cholesterol, 186.50 (IQR = 55.25) mg/dL; and high-density lipoprotein cholesterol (HDL-C), 44.50 (IQR = 16.94) mg/dL. The remaining laboratory parameters are summarized in Table 2.

Correlations between PIIs, symptom severity, functioning, and metabolic parameters

Symptom Severity and Functioning

No significant correlations were observed between any of the PIIs and clinical scores for symptom severity (PANSS), functioning (FAST), or depressive symptoms (CDSS) (Table 3).

**Table 3:** Correlation matrix of peripheral inflammatory indices and clinical variables. Spearman's partial rank correlations were performed to assess the relationship between variables, adjusting for body mass index, age, olanzapine equivalents, and smoking status. AISI: aggregate index of systemic inflammation; CDSS: Calgary Depression Scale for Schizophrenia; FAST: Functioning Assessment Short Test; MLR: monocyte-to-lymphocyte ratio; NLR: neutrophil-to-lymphocyte ratio; PANSS: Positive and Negative Syndrome Scale; PLR: platelet-to-lymphocyte ratio; *r_s_*: Spearman's rho; SII: systemic immune-inflammation index; SIRI: systemic inflammation response index.

Variable		NLR	PLR	MLR	SII	AISI	SIRI
PANSS							
Positive	*r_s_*	0.087	-0.015	0.077	0.010	0.046	0.126
	*p*	0.401	0.883	0.455	0.921	0.655	0.222
Negative	*r_s_*	0.061	-0.042	0.077	0.013	0.037	0.118
	*p*	0.554	0.682	0.454	0.901	0.721	0.252
Cognitive	*r_s_*	0.110	0.016	0.131	0.075	0.117	0.174
	*p*	0.287	0.877	0.204	0.465	0.254	0.090
Excitement	*r_s_*	0.052	-0.013	0.053	0.022	0.028	0.053
	*p*	0.615	0.901	0.606	0.831	0.783	0.605
Anxiety/depression	*r_s_*	-0.099	-0.016	-0.078	-0.122	-0.152	-0.149
	*p*	0.337	0.879	0.453	0.237	0.139	0.147
Total	*r_s_*	0.117	0.053	0.154	0.075	0.106	0.166
	*p*	0.256	0.610	0.133	0.467	0.302	0.107
FAST							
Autonomy	*r_s_*	0.076	0.006	0.016	0.069	0.060	0.073
	*p*	0.459	0.956	0.877	0.505	0.559	0.477
Occupational	*r_s_*	0.080	0.031	0.065	0.119	0.171	0.174
	*p*	0.436	0.763	0.528	0.250	0.096	0.089
Cognitive	*r_s_*	0.154	0.196	0.109	0.128	0.070	0.095
	*p*	0.135	0.055	0.289	0.213	0.500	0.357
Financial	*r_s_*	0.114	-0.019	0.095	0.007	0.035	0.112
	*p*	0.269	0.853	0.357	0.945	0.737	0.275
Interpersonal relationships	*r_s_*	0.119	0.057	-0.055	0.040	-0.043	0.030
	*p*	0.247	0.584	0.592	0.700	0.675	0.772
Leisure	*r_s_*	0.153	0.034	-0.008	0.058	0.000	0.047
	*p*	0.136	0.743	0.939	0.573	1.000	0.646
Total	*r_s_*	0.175	0.087	0.061	0.117	0.084	0.140
	*p*	0.087	0.400	0.555	0.257	0.418	0.172
CDSS	*r_s_*	-0.038	0.047	-0.074	0.001	-0.033	-0.094
	*p*	0.715	0.651	0.472	0.989	0.749	0.363

Metabolic Parameters

Weak positive correlations were observed between HbA1c and SII (*r_s_* (98) = 0.242, *p* = 0.017), AISI (*r_s_* (98) = 0.256, *p* = 0.012), and SIRI (*r_s_* (98) = 0.229, *p* = 0.025). In addition, triglycerides showed weak negative correlations with NLR (*r_s_* (98) = -0.246, *p* = 0.016), MLR (*r_s_* (98) = -0.260, *p* = 0.011), PLR (*r_s_* (98) = -0.256, *p* = 0.012), and SII (*r_s_* (98) = -0.219, *p* = 0.032) (Table 4).

**Table 4:** Correlation matrix of peripheral inflammatory indices and metabolic variables. Spearman's partial rank correlations were performed to assess the relationship between variables, adjusting for body mass index, age, olanzapine equivalents, and smoking status. Values in bold indicate statistical significance at *p* < 0.05. AISI: aggregate index of systemic inflammation; DBP: diastolic blood pressure; HbA1c: glycated hemoglobin; HDL: high-density lipoprotein; MLR: monocyte-to-lymphocyte ratio; NLR: neutrophil-to-lymphocyte ratio; PLR: platelet-to-lymphocyte ratio; *r_s_*: Spearman's rho; SBP: systolic blood pressure; SII: systemic immune-inflammation index; SIRI: systemic inflammation response index.

Variable		NLR	PLR	MLR	SII	AISI	SIRI
Glucose	r_s_	0.168	0.047	-0.300	0.111	0.049	0.071
	p	0.102	0.650	0.774	0.282	0.637	0.493
HbA1c	r_s_	0.134	0.147	0.166	0.242	0.256	0.229
	p	0.192	0.153	0.106	0.017	0.012	0.025
Triglycerides	r_s_	-0.246	-0.256	-0.260	-0.219	-0.169	-0.186
	p	0.016	0.012	0.011	0.032	0.100	0.070
Cholesterol	r_s_	-0.153	-0.151	-0.171	-0.071	-0.032	-0.057
	p	0.138	0.143	0.096	0.493	0.754	0.579
HDL	r_s_	-0.054	0.012	-0.061	0.009	-0.009	-0.037
	p	0.599	0.911	0.556	0.932	0.929	0.721
SBP	r_s_	0.108	0.077	0.015	0.117	0.113	0.045
	p	0.297	0.458	0.883	0.255	0.273	0.660
DBP	r_s_	0.060	0.028	-0.014	0.128	0.151	0.060
	p	0.563	0.788	0.894	0.214	0.142	0.564

## Discussion

This study aimed to evaluate the associations between PIIs and symptom severity, functioning, and metabolic parameters in Mexican patients diagnosed with schizophrenia who were clinically stable and managed under routine clinical care. By adopting a pragmatic approach, we focused on a representative sample of patients in real world clinical setting.

Symptom severity and functioning

Regarding the severity of the disorder, as measured by the five-factor model of the PANSS, no significant correlations were observed with any of the PIIs. This contrasts with previous reports, which have identified positive associations between PIIs, particularly NLR and MLR, and positive, negative, cognitive, and aggressive symptoms [10-13]. However, it is important to mention that the majority of those studies focused on patients with first-episode psychosis or those experiencing acute relapses. In contrast, our sample consisted of clinically stable outpatients with a median disorder duration of 17 years, a median of 36 months on a stable antipsychotic dosage, and without recent hospitalization. Our results suggest that in chronic phases of the disorder, and with the continuous use of antipsychotic treatment, the utility of PIIs in predicting symptom severity is limited. This may be due to the proposed immunomodulatory effects of antipsychotics, which could attenuate systemic inflammation while facilitating symptomatic improvement [14,23]. Consequently, the link between inflammation and symptom severity appears weaker in stable patients than in those in the acute phase. Consistent with this, previous research has shown that NLR values are elevated during acute exacerbations, but not in patients under antipsychotic treatment who are in clinical remission [9].

In terms of psychosocial functioning, no associations were identified with the evaluated indices. To the best of our knowledge, no previous studies have specifically investigated this relationship. Recent evidence has shown that specific cytokines, such as IL-6, are associated with undermentalization, while TNF-α has been linked to poor facial emotion recognition in early psychosis [24]. Given that both processes are fundamental to social cognition, a systemic inflammatory state may contribute to early functional impairment. In contrast to these findings, the level of peripheral innate cellular immunity activation, as reflected by PIIs in this clinically stable and chronic sample, appears insufficient to correlate with the domains of functioning measured by the FAST. Importantly, given that functioning is a complex and multidimensional phenomenon, factors such as other neurocognitive processes (processing speed and attention), negative and depressive symptoms, and social support likely exert a greater influence than the value of PIIs [25,26].

No associations were identified between PIIs and depressive symptoms. To our knowledge, no other studies have specifically aimed to evaluate this in schizophrenia patients. It is important to consider that while increased PIIs (NLR, PLR, and MLR) are well documented in patients with major depressive disorder, our sample only reached a 30% positive screening rate in the CDSS [27]. Additionally, 27% of the participants were receiving antidepressant treatment. This relatively low prevalence of active symptoms, potentially modulated by pharmacological intervention, may explain the lack of a correlation.

Metabolic parameters

We identified weak positive correlations between HbA1c and the SII, AISI, and SIRI. These findings suggest a link between metabolic status and systemic immune activation in schizophrenia. This is consistent with evidence from other populations, where these indices correlate positively with the Homeostatic Model Assessment of Insulin Resistance (HOMA-IR), a validated marker of insulin resistance [28]. A recent study reported a higher level of IL-6 in schizophrenia patients with comorbid diabetes mellitus compared to those without this metabolic condition [29]. Furthermore, research in healthy subjects has shown that proinflammatory cytokines, including TNF-α, promote insulin resistance by altering insulin signal transduction [30]. Notably, IL-6 can also stimulate granulopoiesis in the bone marrow, thus increasing PII values [31]. Collectively, these results suggest that glycemic impairment and systemic inflammation, mediated by both cellular and soluble mediators, are bidirectionally associated in this population. Conversely, we observed weak negative correlations between triglycerides and the NLR, MLR, PLR, and SII. This inverse association may indicate a metabolic-inflammatory decoupling, primarily involving triglycerides, potentially mediated by antipsychotic medication. It is well documented that second-generation antipsychotics can increase triglyceride synthesis while simultaneously exerting immunomodulatory effects that may attenuate the proinflammatory state [4,14,32]. This aligns with previous research suggesting that lower PLR values are predictive of metabolic syndrome [15]. Therefore, these indices may have greater clinical utility as indicators of metabolic risk than of symptom severity or psychosocial functioning. It is important to consider that 21% of our sample were receiving glucose-lowering medications and 12% were on lipid-lowering agents. While this pharmacological intervention may have attenuated the correlations, this patient group more accurately reflects the typical individuals encountered in routine clinical practice.

Strengths and limitations

To our knowledge, this study represents the first research examining the association between PIIs and clinical and metabolic parameters in the Mexican population diagnosed with schizophrenia. Unlike previous studies that used simple ratios, our research employed more complex indices, such as the SII, AISI, and SIRI, which integrate a broader range of innate immune cell lineages to provide a more comprehensive and robust cellular profile than traditional markers. Furthermore, the inclusion of clinically stable patients reduced the potential noise associated with acute psychosis. This is particularly relevant considering that schizophrenia is a dynamic disorder with distinct clinical phases. Additionally, the use of a pragmatic approach, including patients without rigid inclusion criteria and conducted in routine clinical care, enhances the external validity of our study.

Despite these strengths, several limitations must be acknowledged. First, laboratory samples were collected within 12 months prior to the clinical evaluation. The exact timing between blood sampling and clinical assessments was not recorded, and we lack specific data regarding the precise interval for each patient. Therefore, we cannot rule out intercurrent illnesses or medications during this period that may have influenced PII values. Second, although the PANSS is a gold-standard instrument, it may not be sufficiently sensitive to assess specific domains, such as negative symptoms or cognition. Third, our study lacked a healthy control group for the comparison of the PII values between patients and the general population. Fourth, the sample size did not allow for a subgroup analysis by antipsychotic type, an important consideration given that different antipsychotics may have distinct modulatory effects on inflammation. Fifth, we did not account for the potential influence of other pharmacological agents known to modify hematological parameters, such as valproate and lithium, which could impact the interpretation of these indices. Sixth, although our correlations were adjusted for key cofounding factors, including BMI, age, olanzapine equivalents, and smoking status, the observed correlations remained weak. This likely reflects the complex and multifactorial nature of both metabolism and inflammation in schizophrenia. Finally, as our findings suggest that these indices may function as state markers, particularly during acute phases, future research should incorporate longitudinal designs to evaluate PIIs across different clinical stages of schizophrenia.

## Conclusions

In conclusion, our findings suggest that in clinically stable patients and under routine care, PIIs are more associated with metabolic impairment, primarily glycemic and triglyceride alterations, than with symptom severity or psychosocial functioning. In this pragmatic sample of Mexican patients with schizophrenia, these indices may serve as accessible and cost-effective candidate immunometabolic indicators. Given the high prevalence of metabolic syndrome and its associated cardiovascular risk in this population, it is essential to further investigate these indices to evaluate their utility in metabolic risk assessment and the possibility of incorporating them as auxiliary tools for psychiatrists. Also, future longitudinal studies are necessary to determine the predictive value of PIIs across different clinical phases of the disorder.
